# Mirogabalin Eliminated Pain and Reduced Inflammation in Visceral Pain

**DOI:** 10.33549/physiolres.935613

**Published:** 2026-02-01

**Authors:** Isa YESILYURT, Soner BITIKTAS, Serdar YIGIT, Basak GULAKAR, Huseyin FATIH GUL

**Affiliations:** 1 Department of Physiology, Faculty of Medicine, Yalova University, Yalova, Republic of Türkiye; 2 Department of Physiology, Faculty of Medicine, Kafkas University, Kars, Republic of Türkiye; 3 Department of Histology and Embryology, Faculty of Medicine, Kafkas University, Kars, Republic of Türkiye; 4 Department of Medical Biochemistry, Faculty of Medicine, Kafkas University, Kars, Republic of Türkiye

**Keywords:** Mirogabalin, Pain, Inflammation, Visceral, GABA

## Abstract

Mirogabalin is a newly developed gabapentinoid drug. Several *in vivo* and clinical studies have demonstrated the potent analgesic effects of mirogabalin in neuropathic pain. This study aims to investigate the impact of mirogabalin on visceral pain and inflammation. Adult male Balb/c mice (20–25 g) were used in the study (n=7). Mirogabalin was administered intraperitoneally at 10, 20, and 40 mg/kg doses. Inflammatory visceral pain was induced by intraperitoneal administration of acetic acid. The number of writhing was observed after acetic acid administration, and the effective dose of mirogabalin was determined. In the second phase of the study, the effects of mirogabalin on locomotor activity and leukocyte infiltration into peritoneal tissue were examined. IL-6, GSH levels, and SOD activity were investigated biochemically. Statistical analyses were performed in the GraphPad Prism (v8.0.1) program. Mirogabalin was significantly antinociceptive at all three doses (p<0.001). Histopathologic examination showed that the effective dose of mirogabalin decreased leukocyte infiltration into the peritoneum. Mirogabalin did not affect total distance moved and mean speed in the open field test. There was no significant difference between the groups in terms of IL-6, GSH levels, and SOD activity. Our results demonstrated a significant antinociceptive effect of mirogabalin against visceral pain. In addition, anti-inflammatory effects were revealed by decreasing leukocyte infiltration. However, the fact that mirogabalin did not alter antioxidant systems and IL-6 levels suggests that other mechanisms are responsible for its anti-inflammatory effects.

## Introduction

Calcium is an ion involved in many essential physiological processes. Intracellular calcium entry *via* voltage-gated calcium channels (VGCCs) triggers numerous intracellular events such as muscle contraction, vesicle fusion for neurotransmitter and hormone release, gene transcription, programmed cell death, and activation of intracellular signaling cascades. N-type VGCCs are frequently found in pain pathways, and some blockers of these channels are used in various pain-related clinical cases [1,2]. However, pain treatment varies significantly due to factors such as the tissue of origin, etiology, and duration [3]. In addition, current treatment modalities can lead to significant side effects. Therefore, preclinical pain studies are still necessary. N-type VGCCs are mainly found in the presynaptic nerve terminal. N-type VGCCs are abundant in the dorsal root ganglion, a region crucial in pain transmission, and are involved in neurotransmitter release [4]. Therefore, drugs that affect N-type VGCCs are expected to have potential in pain treatment. Antinociceptive effects of mirogabalin, an N-type VGCC blocker, have been reported in experimental pain models [5,6]. The gabapentinoid group of medicines, including mirogabalin, are used for neuropathic pain, and mirogabalin was approved for the treatment of this indication in 2019 [7].

While the anti-inflammatory effects of other gabapentinoid drugs have been demonstrated, mirogabalin has been poorly studied in this regard [8]. A recent study revealed the antinociceptive effects of mirogabalin in the formalin test, a model of inflammatory pain [5]. However, in that study, no evaluation was made regarding inflammation markers. In another study, the effect of mirogabalin on proinflammatory cytokines involved in the development of the neuropathic pain model was investigated, but mirogabalin did not change cytokine levels [6].

Based on these data, it is understood that there are no studies on the effects of mirogabalin on visceral pain and insufficient data on its impact on inflammation. Visceral pain differs from somatic pain as it is not well localized, causes reflected pain and causes autonomic and motor reflexes more frequently. However, the majority of information on pain has been obtained from somatic pain studies.

This study aimed to investigate the effects of mirogabalin on the inflammatory visceral pain model (the writhing test), inflammation, and locomotor activity.

## Methods

Adult male Balb/c mice (20–25 g) were used in the study. Female mice were omitted because the pain response may change during different periods of estrus. The experimental animals were kept under standard laboratory conditions (22±1 °C room temperature, 12 h light/dark cycle, and free access to feed and water) before the experiments.

All experiments were approved by the Kafkas University Local Ethics Committee for Animal Experiments numbered 2024/185. The studies were conducted in accordance with the Guide for the Care and Use of Laboratory Animals and the ethical guidelines of the International Association for Pain Research (IAPS).

No surgical procedure was performed on the animals before the experiments, and the experiments were performed in a single laboratory between 08:00 and 16:00 h for standardization.

The study by Bagheri *et al.* served as a reference for estimating the sample size to be used in the present research [9]. A statistical power analysis was conducted with the G*Power program (version 3.1.9) to determine the minimum number of animals needed. According to the analysis outcomes, each group was assigned seven animals. A total of 56 experimental animals, 7 in each group, were used. After the end of the behavioral experiments, the animals were anesthetized with intraperitoneal (i.p.) ketamine (90 mg/kg) and xylazine (10 mg/kg) and then sacrificed by cervical dislocation.

In the study's first phase, a dose-response analysis of mirogabalin in the writhing test was performed. In the sham group, acetic acid and DMSO (2.5 %), the solvent of mirogabalin, were administered. In the dose-response groups, mirogabalin was administered i.p. at 10, 20, and 40 mg/kg doses half an hour before acetic acid, and the effective dose was investigated. In the second phase of the study, control, sham, effective mirogabalin dose group, and acetic acid+mirogabalin groups were included.

### Drugs

Mirogabalin (Aobious INC, Gloucester, USA), an N-type VGCC selective α2δ-1 ligand, was used in the study. Dose-response studies were performed with three doses of pregabalin based on the doses that are effective in various pain models in the literature. Mirogabalin was administered i.p. at 10, 20, and 40 mg/kg doses. Mirogabalin was dissolved in 2.5 % DMSO. All i.p. injections were made in a volume of 0.1 ml/10 g.

### Acetic Acid Writhing Test

The writhing test was used to induce visceral pain in mice. Mice were administered 0.6 % acetic acid solution in a volume of 0.1 ml/10 g i.p. The characteristic writhing behavior (N) was observed and counted for 15 min starting from the 5^th^ minute after acetic acid administration.

### Histological analysis

Following the end of the observation period in the writhing test, the animals were anesthetized with ketamine (90 mg/kg, i.p.) and xylazine (10 mg/kg, i.p.) and then sacrificed by cervical dislocation. After peritoneum were removed, they were fixed in 10 % formalin solution for 72 h and tissue follow-up was performed according to the literature. After tissue follow-up, 5 micrometer thick serial sections were taken from each paraffin block for histopathologic examinations. Hematoxylin-eosin (H&E) staining was performed. Photographs were taken on an Olympus BX43 microscope using the Cellsense Software program.

### Assessment of locomotor activity

An open-field test investigated whether the effective dose of mirogabalin affects locomotor activity. The open-field test is a commonly used method to examine the effect of drugs on locomotor activity in various pain and inflammation studies. For this purpose, a 40×40×40 cm platform was used. The mice were placed in the center of the platform, and the distance moved, average speed, and time spent in the center and periphery were observed using a video camera system. Recording began 35 min after drug administration, in accordance with the timing of the pain experiments, and lasted for 5 min. Video recordings were analyzed using the open-source ToxTrac software (Open Behaviour, USA). The effective dose of mirogabalin was compared with the control group.

### Biochemical analyses

#### Preparation of liver tissue homogenates

The cryopreserved mouse-liver tissue samples were accurately weighed and subjected to homogenization in ice-cold 0.01 M phosphate-buffered saline (PBS, pH 7.4) at a ratio of 1/10 (w/v) using a programmable tissue homogenizer (Ultra Turrax T25-B, IKA Labortechnik) set at 3,000 rpm for 20 min at a controlled temperature of 4 °C. Mouse liver tissue homogenates were centrifuged at 10,000× g for 10 min at 4 °C. The supernatants were collected into Eppendorf tubes and after centrifugation, the clear supernatants were immediately processed.

#### Measurement of Interleukin 6 (IL-6) and Reduced Glutathione (GSH) levels in liver tissue

The IL-6 and GSH levels in clear supernatants were analyzed using the Enzyme-Linked Immunosorbent Assay (ELISA) protocol outlined in the manufacturer's datasheet. The quantifications of Interleukin 6 (IL-6) and reduced glutathione (GSH) levels in liver tissue were carried out using the Mouse Interleukin 6 (IL-6) ELISA Kit (Catalog no 201-02-0050, lot no 202403, Exp date;2025/03, Shanghai, China) and Mouse Reduced Glutathione (GSH) ELISA Kit (Catalog no 201-02-0180, lot no 202403, Exp date 2025/03, Shanghai, China) as per the manufacturer's instructions. Tissue samples were prepared in duplicate and results were performed using the ELISA instruments mentioned above. The concentrations were calculated based on standard curves. The tissue IL-6 and GSH concentrations were expressed as nanograms per gram protein (ng/g protein) and milligrams per gram protein (mg/g protein), respectively, taking into account the dilution factor and tissue total protein ratios. Total protein concentration was measured concentration was performed using the method described by Lowry *et al.* [10,11].

#### Measurement of Total Superoxide Dismutase (T-SOD) activity in mouse liver tissue

The T-SOD activity of clear supernatants of tissue samples was measured by using Elabscience Total Superoxide Dismutase (T-SOD) Activity Assay Kit (Hydroxylamine Method, Cat. No: E-BC-K019-M, Lot No: WO17V44X7817 Exp (MM/DD/YY):04/11/2025, Houston, TTX77079, USA) the manufacturer's datasheet. Tissue samples were prepared in duplicate and results were performed using the ELISA instruments mentioned above. The T-SOD activity was calculated indicated on the kit's datasheet formula. T-SOD activity of liver tissue samples is expressed as U/mg protein. The dilution factor of the tissue samples was set to 160 as recommended by the kit in the table. Performed. Total protein concentration was measured using the method described as above. The performance values of the kit are as stated above.

#### Statistical analysis

All statistical analyses were performed in the GraphPad Prism v8.0.1 program. Results are expressed as mean ± standard error of the mean. Shapiro Wilk test was applied to evaluate the conformity of the data to normal distribution. Independent *t*-test and one-way analysis of variance (ANOVA) were used to analyze the groups fitting the normal distribution, followed by the Tukey Kramer *post hoc* test for multiple group comparisons. p<0.05 was considered statistically significant.

## Results

### Effects of Mirogabalin on pain, inflammation and locomotor activity

**Fig. 1:**
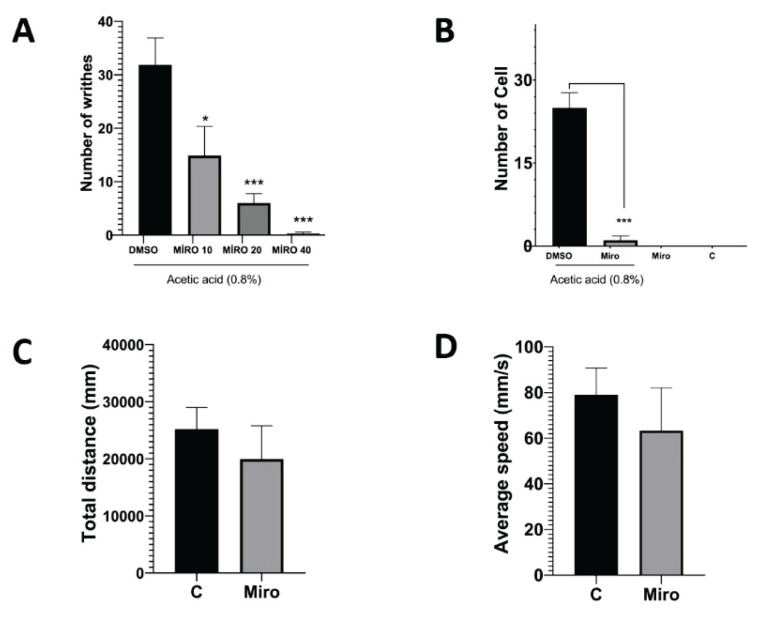
Effects of mirogabalin on nociceptive, behavioral, and histopathological parameters. (**A**) Number of writhes in the acetic acid-induced writhing test representing pain behavior. (**B**) Leukocyte infiltration in peritoneal tissue determined by histopathological analysis. Total distance moved (**C**) and average speed (**D**) measured in the open field test reflecting locomotor activity. Mirogabalin was administered at a dose of 20 mg/kg (i.p.) in the experiments shown in Figures 1B, 1C, and 1D.

Mirogabalin was administered 30 min before acetic acid in the writhing test at doses of 10, 20, and 40 mg/kg i.p., respectively. In the sham group, 2.5 % DMSO, the solvent of mirogabalin, was administered 30 min before acetic acid. Mirogabalin showed a significant antinociceptive effect at all three doses (Fig. 1A). Pain behavior decreased by 53.36 %, 81.17 %, and 99.2 % in the mirogabalin dose groups. p values were 0.0238, 0.0008, and <0.0001, respectively. For histopathologic examination and open field tests, 20 mg/kg i.p. was determined as the effective dose of mirogabalin.

**Fig. 2:**
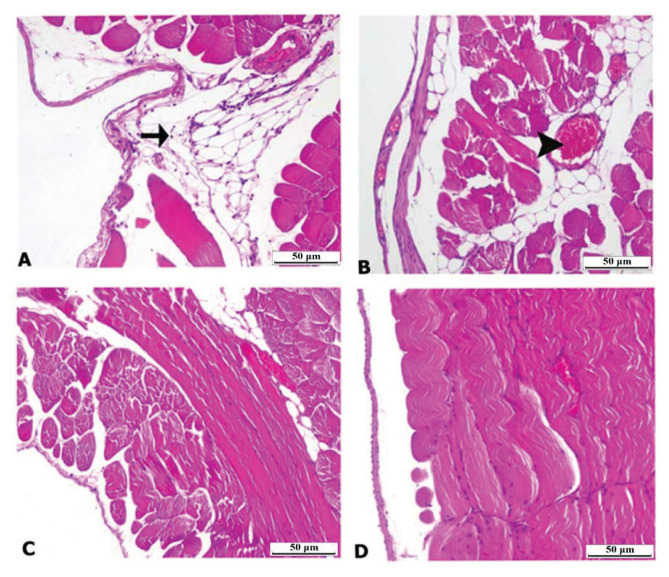
Effect of mirogabalin (20 mg/kg i.p.) on leukocyte infiltration in peritoneal tissue. (**A**) Acetic acid administered, (**B**) Miro (20 mg/kg i.p.) + acetic acid administered, (**C**) Miro (20 mg/kg i.p.) alone, (**D**) control groups.

In histopathologic analysis of peritoneal tissue, intense leukocyte cells were observed in the acetic acid-administered group. Leukocyte cell numbers were significantly lower in the mirogabalin-treated group 30 min before acetic acid (Fig. 1B and Fig. 2) (p<0.001).

In the open field test, no significant difference was observed between the mirogabalin and control groups regarding total distance moved and average speed (Fig. 1C, 1D). p values were 0.0678 and 0.0843, respectively.

### Effect of Mirogabalin on tissue IL-6, SOD and GSH levels

**Fig. 3:**
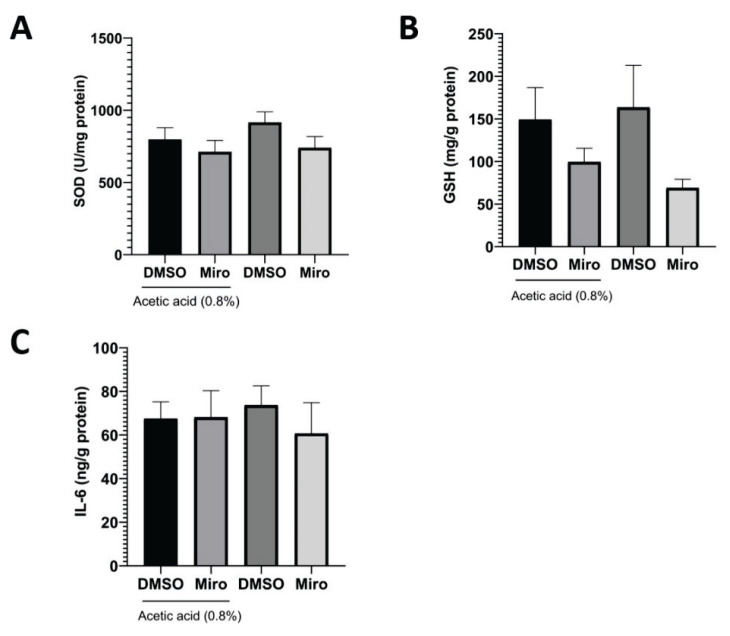
Effect of Mirogabalin (20 mg/kg i.p.) on IL-6 (**A**), GSH (**B**) levels and SOD activity (**C**).

Tissue IL-6 and GSH levels were investigated using the ELISA method in tissue samples obtained from mouse livers. No significant difference was found between the groups regarding IL-6 levels (p=0.89). Although the lowest IL-6 level was observed in the mirogabalin group, this difference was not statistically significant. Based on the ELISA analysis, there was no significant difference between the groups regarding tissue GSH levels (p=0.20) (Fig. 3A, 3B). SOD activity levels were measured in liver tissue samples obtained from the experimental groups. No significant difference was observed between the groups in terms of SOD activity (p=0.92) (Fig. 3C).

## Discussion

Our study showed that mirogabalin exhibited significant antinociceptive effects at 10, 20, and 40 mg/kg i.p. doses in the visceral pain model. In particular, at 40 mg/kg dose, almost no pain behavior was observed in animals. Mirogabalin also significantly reduced inflammation in histopathologic examination. However, biochemical analysis revealed that mirogabalin did not exert this effect through the oxidant/antioxidant system.

Gabapentinoids are a group of drugs classified under calcium channel blockers [12]. Gabapentinoids are widely used in pain treatment due to their safety compared to opioids. Specifically, neuropathic pain, headache, and skeletal-muscular pain are among the most prominent indications for gabapentinoids [13]. Their mechanisms of action have yet to be fully understood. Although this group of drugs is related to GABA, they do not interact directly with GABA receptors [14]. Gabapentinoids slightly increase the concentration of GABA in the brain [15]. However, the antinociceptive effects of gabapentinoids are responsible for their binding to α2δ subunits of VGCCs and reducing the secretion of excitatory neurotransmitters [13]. Similar to gabapentin-noids, ziconotide, a synthetic N-type VGCC blocker, has been found to have potent efficacy similar to opioids in the treatment of pain by intrathecal infusion [16].

While the antinociceptive effects of gabapentinoids are mainly related to their reduction of neurotransmitter secretion, inflammation-reducing effects of these drugs have also been demonstrated. A study by Camara *et al.* found that gabapentin administration decreased inflammatory cell migration and inflammation-related cytokines in carrageenan-injected Wistar rats with chronic constriction injury [8]. A study conducted on human volunteers with inflammatory pain similarly demonstrated the anti-inflammatory effects of gabapentin [17]. Pregabalin has also shown anti-inflammatory effects like gabapentin. A study by Kılıç *et al.* showed that pregabalin decreased acute inflammation and proinflammatory cytokine levels [18]. In another study, inflammation was induced by lipopolysaccharide in albino Wistar rats, and it was observed by histopathologic, biochemical, and immunohistochemical methods that pregabalin administration decreased inflammation [19].

Mirogabalin, a new gabapentinoid derivative, inhibits VGCCs like other group members [20]. Following its development, mirogabalin was immediately investigated in experimental neuropathic pain studies, and its antinociceptive effects were demonstrated [5,6,21,22]. Mirogabalin has also shown efficacy in patients with diabetic peripheral neuropathic pain and spinal cord injury [23,24]. There is limited data on the effects of mirogabalin on other types of pain and inflammation. No other study has been conducted on the impact of mirogabalin in an inflammatory visceral pain model until our study. In a 2021 study, mirogabalin showed an antinociceptive effect in the formalin test, an acute inflammatory pain model [5]. Nevertheless, the effect of mirogabalin on inflammation was not investigated in that study. In another study in which sciatic nerve injury was induced in mice, administration of mirogabalin showed a higher antinociceptive effect than pregabalin. In the same research, it was found that mirogabalin decreased microglia migration and affected cytokines involved in the neuropathy pathway [6]. Gabapentinoids have been found to reduce inflammatory cell migration [19]. These findings are consistent with our histopathologic results. However, our biochemical analysis showed that mirogabalin did not affect reactive oxygen species and proinflammatory cytokines. In a recent study, chronic constriction injury was induced in mice, and various signaling molecule levels were examined. In mice with neuropathy, mirogabalin administration did not change IL-1β and IL-6 levels but decreased substance P mRNA levels [6]. These data confirm that inhibition of neurotransmitter release is responsible for the main antinociceptive effect of mirogabalin. However, as mentioned above, the leukocyte migration-reducing effect is noteworthy.

According to our open-field test findings, mirogabalin did not affect locomotor activity. The same test was used in another study using trigeminal nerve injury in mice, and similar results were obtained [25]. There were also studies with different findings regarding the effect of mirogabalin on locomotor activity. However, the possible reasons for this situation are the various types of experimental animals and mirogabalin doses [5].

One of the most valuable features of our study is that the effect of mirogabalin, a newly developed drug, was investigated in an inflammatory visceral pain model for the first time. In addition, the histopathologic demonstration of peritonitis in the writhing test is a novelty. This feature is a candidate for guiding pain researchers who will study similar models in the future. The limitations of our study include the lack of molecular analyses demonstrating protein expression and neurotransmitter secretion levels. Specifically, we did not evaluate the expression of key markers such as α2δ-1, substance P, or proinflammatory cytokines in the dorsal root ganglia or in the peritoneal tissue where inflammation was induced. Future studies should address these limitations by investigating molecular changes in inflammation-related and pain-processing tissues to clarify the mechanisms underlying mirogabalin’s anti-inflammatory effects.

After this study, it is necessary to reveal the mechanism of action of mirogabalin, which we have observed to have potent antinociceptive effects. New studies using molecular methods involving receptors and second messenger pathways seem possible in this respect.
